# Preoperative diagnostic categories of fine needle aspiration cytology for histologically proven thyroid follicular adenoma and carcinoma, and Hurthle cell adenoma and carcinoma: Analysis of cause of under- or misdiagnoses

**DOI:** 10.1371/journal.pone.0241597

**Published:** 2020-11-04

**Authors:** Hee Young Na, Jae Hoon Moon, June Young Choi, Hyeong Won Yu, Woo-Jin Jeong, Yeo Koon Kim, Ji-Young Choe, So Yeon Park

**Affiliations:** 1 Department of Pathology, Seoul National University Bundang Hospital, Seoul National University College of Medicine, Seoul, Republic of Korea; 2 Department of Internal Medicine, Seoul National University Bundang Hospital, Seoul National University College of Medicine, Seoul, Republic of Korea; 3 Department of Surgery, Seoul National University Bundang Hospital, Seoul National University College of Medicine, Seoul, Republic of Korea; 4 Department of Otorhinolaryngology–Head and Neck Surgery, Seoul National University Bundang Hospital, Seoul National University College of Medicine, Seoul, Republic of Korea; 5 Department of Radiology, Seoul National University Bundang Hospital, Seoul National University College of Medicine, Seoul, Republic of Korea; 6 Department of Pathology, Hallym University Sacred Heart Hospital, Anyang, Republic of Korea; Universidade do Porto Faculdade de Medicina, PORTUGAL

## Abstract

Cytologic diagnosis of thyroid follicular adenoma and carcinoma, and Hurthle cell adenoma and carcinoma (FACHAC) is challenging due to cytomorphologic features that overlap with other follicular-patterned lesions. This study was designed to analyze diagnostic categories (DCs) of preoperative fine needle aspiration cytology (FNAC) of histologically proven thyroid FACHACs to evaluate under- or misdiagnoses in FNAC and elucidate potential causes for such phenomena. A total of 104 thyroid nodules with preoperative FNAC which were diagnosed as FACHAC in resection specimens were included in this study. Of these, 66 cases had also undergone thyroid core needle biopsy (CNB); FNAC and CNB DCs were compared in these cases. Various cytologic and histologic parameters were compared between the nodules with different FNAC DCs. After a review of FNAC slides, DCs were re-assigned in 20 (19.2%) out of the 104 cases. Of the 66 cases with CNB diagnoses which were mostly classified as lower DCs in FNAC, 31 (47.0%) were diagnosed as suspicious for a follicular neoplasm in CNB. Cases which were underdiagnosed in FNACs were associated with lower cellularity, predominant macrofollicular pattern, absence of microfollicles arranged in trabecular pattern, and absence of transgressing vessels in cytology smears. High cellularity, microfollicles arranged in trabecular pattern, nucleolar prominence, and large cell dysplasia were more frequently found in malignancy than in benign neoplasm. In conclusion, thyroid FACHACs seem to be under- and misdiagnosed in preoperative FNAC. Innate characteristics of the nodules were associated with under-diagnosis as well as the quality of the FNAC specimens. Certain cytomorphologic features can be helpful in differentiating malignancy among FACHACs.

## Introduction

Ultrasonography (USG)-guided fine needle aspiration cytology (FNAC) is the most commonly used preoperative testing method for thyroid nodules. Currently, FNAC results are classified into six diagnostic categories (DCs) according to The Bethesda System for Reporting Thyroid Cytopathology (TBSRTC) in most countries [1, 2]. Although thyroid FNAC is diagnostic in a majority of benign nodules as well as in most papillary thyroid carcinomas (PTCs) and other types of carcinomas, it generally functions as a screening test for follicular-patterned lesions.

The cytologic findings of follicular adenoma (FA) and carcinoma, and Hurthle cell adenoma (HA) and carcinoma [FACHAC] can significantly overlap with various other thyroid follicular-patterned lesions including nodular hyperplasia, noninvasive follicular thyroid neoplasm with papillary-like nuclear features (NIFTP), and even follicular variant PTC [3–5]. In addition, a final diagnosis of follicular thyroid carcinoma (FTC) or Hurthle cell carcinoma (HC) can only be made after thorough examination and confirmation of capsular and/or vascular invasion in the resection specimen. Although TBSRTC suggests certain criteria for rendering DC IV (suspicious for a follicular neoplasm; SFN) to identify potential FTCs or HCs and to refer them for diagnostic lobectomy with higher sensitivity rather than higher specificity [1, 2], preoperative cytologic diagnosis of FACHAC remains challenging. The incidence of FTC is much lower than PTC in Korea [6], however, there has been an increase in detection rates of indeterminate nodules through screening USG of the thyroid [6–8]. Since FTC and HC can potentially progress to distant metastasis, it is important not to underdiagnose or misdiagnose these malignancies and prevent the treatment delay in patients with these tumors.

USG-guided thyroid core needle biopsy (CNB) has been continuously reported to be a useful complementary tool for FNAC by reducing non-diagnostic or indeterminate results, especially when the results are reported according to a standardized system [9–12]. Moreover, CNB has been reported to be a more reliable method than FNAC in diagnosing follicular-patterned neoplasm with lower false positive rates, and higher risk of malignancy rates [13–15]. This advantage of CNB over FNAC is attributed to the fact that CNB can provide histologic information including not only the nodule itself, but also its relationship with the capsule and surrounding normal thyroid tissue [13–15].

According to the nationwide survey done by the Korean Society for Cytopathology in 2012, the average rate of TBSRTC DC IV (SFN) was 0.9% (range 0–2.1%) [16], a number much smaller compared to western countries [17]. Thus, in the present study, we reviewed preoperative FNACs of thyroid nodules with final surgical diagnoses of FACHAC to investigate whether there were under- or misdiagnoses in FNAC. In cases in which preoperative CNB had been performed, we compared DCs of FNAC and CNB. Finally, we analyzed various cytologic and histologic features of each nodule and correlated them with FNAC DCs to identify the potential causes of under- or misdiagnoses.

## Materials and methods

### Cases selection

We collected a total of 11,695 thyroid FNACs from 10,824 patients diagnosed at Seoul National University Bundang Hospital from January 2012 to December 2018. Of 10,824 patients, 270 patients underwent repeated FNACs for the same nodule, and the DC with the highest risk of malignancy was selected. In 592 patients, multiple nodules were separately aspirated, and they were considered individual cases. Finally, 11,396 FNAC cases were used for this study. All FNAC slides were diagnosed according to TBSRTC, 1^st^ or 2^nd^ edition [1, 2].

Of the 11,396 FNACs, a total of 4,369 nodules were surgically resected, yielding 190 (4.3%) cases of FACHAC; 102 (2.3%) FAs, 42 (0.9%) FTCs, 39 (0.9%) HAs, and 7 (0.2%) HCs. Of these 190 cases, 121 cases had both FNAC and surgical resection slides available. We re-evaluated surgical slides of these 121 cases according to the 2017 WHO classification [18], and 17 cases showing unequivocal nuclear atypia (nuclear score 2 or 3) [19] were re-classified as PTCs: 2 encapsulated variant PTCs with predominant follicular pattern and 15 invasive encapsulated follicular variant PTCs. Finally, a total of 104 cases with final diagnoses of FACHAC were included in the present study. This study was approved by the Institutional Review Board (IRB) of Seoul National University Bundang Hospital (IRB No. B-2003-598-301), and the requirement for informed consent was waived. All the samples used in this study were obtained from archival material in the Department of Pathology. All the data including patient record and samples were fully anonymized before we analyzed them.

### Ultrasonography and USG-guided FNAC and CNB procedures

Thyroid USG (iU22, Philips Medical Systems, Bothell, WA), USG-guided thyroid FNACs, and CNBs were performed by one of three board-certified radiologists. FNACs were done using a 22- to 25-gauge needle. CNBs were executed using 18-gauge automatic biopsy needles with a 1.1-cm excursion (TSK Ace-cut; Create Medic, Yokohama, Japan). One to two cores of specimen were obtained for each thyroid nodule. As previously described [10], CNBs were performed after considering the size and imaging features of the thyroid nodules including (1) any suspicious malignant nodule (with any one of the following features: taller than wide shape, spiculated margin, marked hypoechoicism, microcalcification, or macrocalcification), over 5 mm; (2) an indeterminate nodule (without probably benign features or suspicious malignant features), over 10 mm; and (3) a probably benign nodule (isoechoic spongiform nodule, comet-tail artifact, predominantly cystic) over 20 mm, according to the 2009 ATA management guidelines [20] and the consensus statement of the Korean Society of Thyroid Radiology [21].

### Review of FNAC

**Fig 1:**
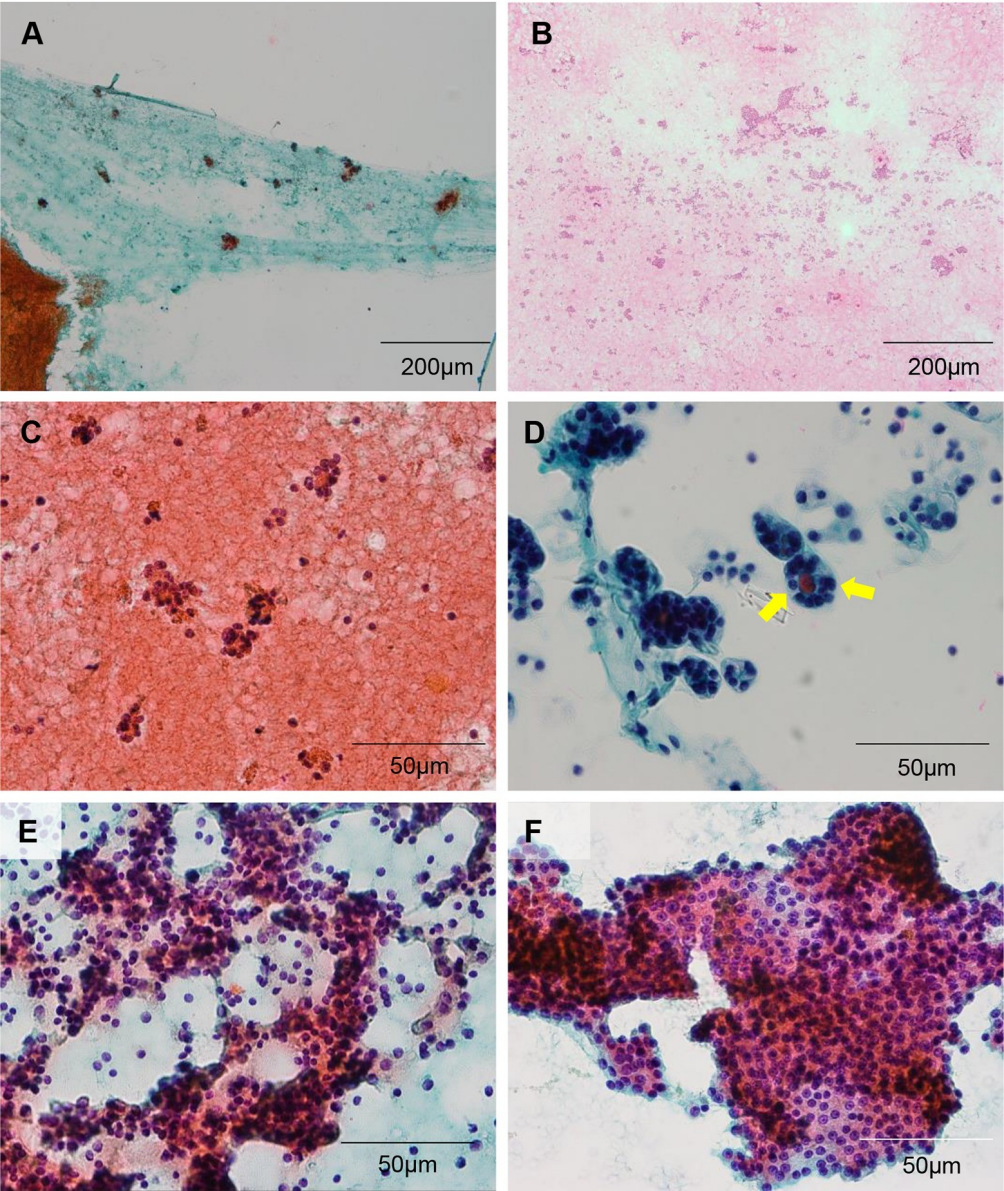
Cytologic features of histologically proven follicular adenoma and carcinoma, and Hurthle cell adenoma and carcinoma. (A) A representative case classified as diagnostic category (DC) III (atypia of undetermined significance) showing sparsely cellular specimen (x15; scale bar, 200 μm). (B) A case diagnosed as DC IV (suspicious for a follicular neoplasm) shows moderately cellular specimen with abundant microfollicles (x15; scale bar, 200 μm) (C-F) Architectural alterations such as microfollicles (C and D), 3-dimensional branching (E), and architectural crowding (F) are frequently observed in cases categorized as DC IV (suspicious for a follicular neoplasm). In some cases, thick colloid (D) is noted within microfollicles (arrows) (x200; scale bar, 50 μm).

**Fig 2:**
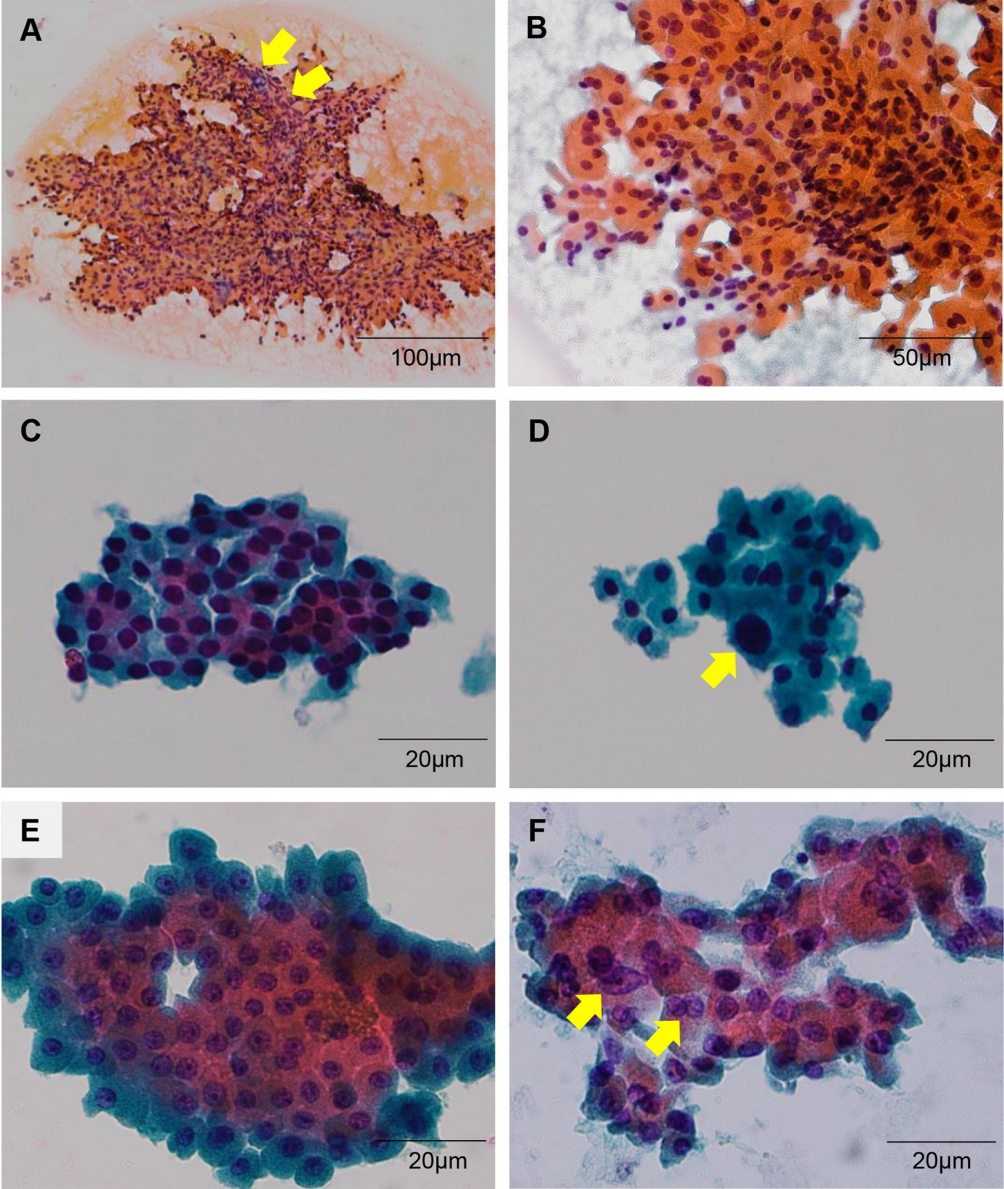
Cytologic features of histologically proven Hurthle cell adenoma and carcinoma. (A) Transgressing vessels are common in both Hurthle cell adenoma and carcinoma (x100; scale bar, 100 μmμ). (B) Cells generally show hyperchromatic nuclei with abundant granular cytoplasm (x200; scale bar, 50 μm). (C-F) Small cell dysplasia (C), large cell dysplasia (D), and prominent nucleoli (E) are seen in some cases. Importantly, focal chromatin clearing and nuclear groove (F) could be observed, features of which, can lead to misdiagnosis (x400; scale bar, 20 μm).

**Fig 3:**
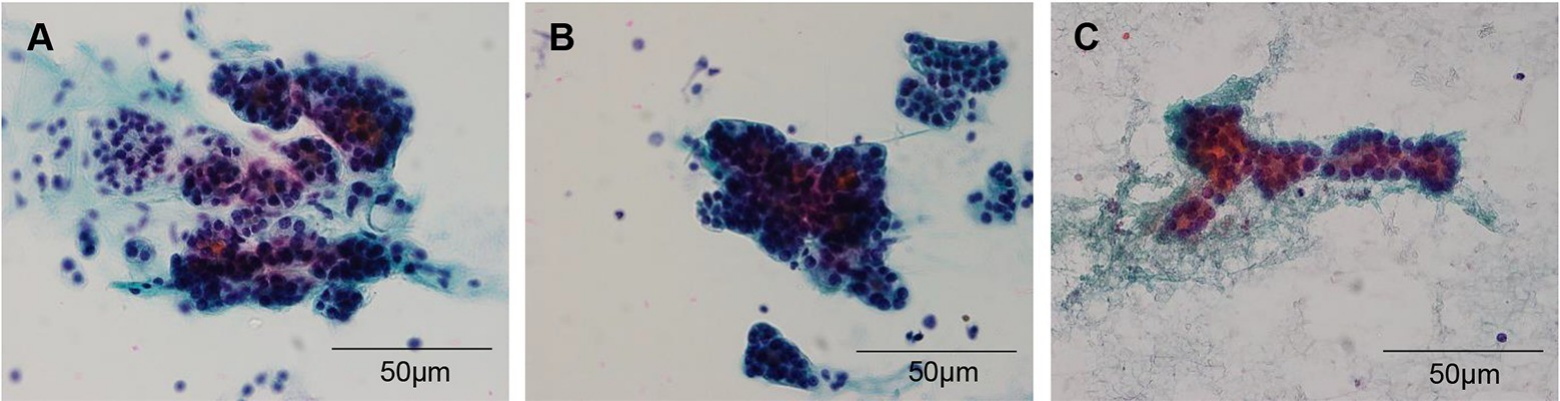
Representative images of microfollicles arranged in trabecular pattern. (A-C) Microfollicles arranged in trabecular (A and B) or branching patterns (C) are predominantly observed in follicular thyroid carcinoma (x200; scale bar, 50 μm).

All thyroid FNAC slides were reviewed by two experienced pathologists (SYP and HYN) in a blind manner. Discordant cases were discussed to reach consensus. The diagnoses were made according to TBSRTC, 2^nd^ edition [2]. Various cytologic parameters including cellularity, presence of artifact, architectural pattern, nuclear features, and background quality were analyzed in FNAC specimens. As for architectural pattern, proportions of macrofolliclular- and microfollicular patterns were evaluated. In addition, types of architectural alteration reported in FN and Hurthle cell neoplasm (HN) were analyzed: the presence of architectural crowding, 3-dimensional branching pattern, microfollicles arranged in trabecular pattern, and transgressing vessels (Figs 1 and 2). Of these, “microfollicles arranged in trabecular pattern” was defined as microfollicles forming 3-dimensional clusters and trabecular arrangement as described in a previous study by Han et al [22] (Fig 3). Regarding nuclear features, the presence of nuclear enlargement, anisonucleosis, nuclear hyperchromasia, prominent nucleoli, small cell or large cell dysplasia, and the presence of PTC-like nuclear atypia was described. Small cell dysplasia was defined as the cytoplasmic diameter less than twice the nuclear diameter, and large cell dysplasia as the cytoplasmic diameter greater than twice the variation in nuclear diameter [23] (Fig 2). Presence of background colloid, blood, or cystic change was also recorded. When colloid was present, the type of colloid (thin, thick, and both) was also noted.

### Pathologic review of CNB specimens

Of the 104 cases, 66 cases were also evaluated by thyroid CNB. The DCs of thyroid CNB were compared with those of FNAC. The diagnosis of CNB was made into one of six DCs based on the standardized reporting system proposed by the Korean Endocrine Pathology Thyroid Core Needle Biopsy Study Group. The six DCs are similar to those of TBSRTC; I. non-diagnostic, II. benign lesion, III. indeterminate lesion, IV. SFN, V. suspicious for malignancy, and VI. malignant. Indeterminate lesion was subdivided into indeterminate lesion with architectural atypia and indeterminate lesion with nuclear atypia. SFN category was subdivided into SFN without nuclear atypia and SFN with nuclear atypia [24]. All thyroid CNB slides were reviewed by two pathologists (SYP and HYN) in a blind manner, and discordant cases were discussed to reach consensus.

### Pathologic review of resection specimens

To identify whether the cytologic diagnoses are affected by their histologic features, we analyzed various histologic parameters of tumors in resection specimen; proportion of normo- and macrofollicular pattern, papillary hyperplasia, intratumoral fibrosis, calcification or bony metaplasia, cystic degeneration, hemorrhage, background lymphocytic thyroiditis and tumor size. All resection specimens were also reviewed by two pathologists (SYP and HYN) in a blind manner, and discussion for the discordant cases was performed.

### Statistical analyses

Statistical analyses were performed by using SPSS version 22.0 (IBM, NY, USA). To compare the frequencies of categorical variables between two groups, Pearson chi-square test or Fisher’s exact test were applied. All p-values reported were two-sided, and a p-value of less than 0.05 was considered statistically significant.

## Results

### Preoperative FNAC diagnostic categories of histologically proven FACHAC

The original DCs of 104 FNAC cases were retrieved from the electronic medical records. All slides were carefully reviewed in a blinded fashion. DCs were revised in 20 out of the 104 cases. In the original diagnoses, 18 (17.3%), 13 (12.5%), 63 (60.6%), 7 (6.7%), 2 (1.9%), and 1 (1.0%) were classified as DC I (non-diagnostic), II (benign), III (AUS), IV (SFN), V (suspicious for malignancy), and VI (malignant), respectively (Table 1). After review, 20 (19.2%), 8 (7.7%), 60 (57.7%), and 16 (15.4%) cases were categorized into DC I (non-diagnostic), II (benign), III (AUS), IV (SFN), respectively (Table 2). Detailed cytologic and histologic features of FACHACs included in the current study are summarized in S1 Data.

**Table 1:** Original thyroid FNAC diagnostic categories of histologically proven follicular adenoma and carcinoma, and Hurthle cell adenoma and carcinoma.

FNAC diagnostic category	No. of surgical specimens	Final diagnosis			
		FA	FTC	HA	HC
I. Non-diagnostic	18 (17.3%)	9 (21.4%)	3 (12.5%)	6 (18.8%)	0 (0%)
II. Benign	13 (12.5%)	4 (9.5%)	4 (16.7%)	5 (15.6%)	0 (0%)
III. AUS	63 (60.6%)	25 (59.5%)	14 (58.3%)	20 (62.5%)	4 (66.7%)
AUS-CA	19 (18.3%)	9 (21.4%)	4 (16.7%)	6 (18.8%)	0 (0%)
AUS-AA	21 (20.2%)	12 (28.6%)	8 (33.3%)	1 (3.1%)	0 (0%)
AUS-CA and AA	4 (3.8%)	2 (4.8%)	2 (8.3%)	0 (0%)	0 (0%)
AUS-H	19 (18.3%)	2 (4.8%)	0 (0%)	13 (40.6%)	4 (66.7%)
IV. Suspicious for a FN	7 (6.7%)	3 (7.1%)	3 (12.5%)	1 (3.1%)	0 (0%)
V. Suspicious for malignancy					
suspicious for papillary carcinoma	2 (1.9%)	1 (2.4%)	(0%)	0 (0%)	1 (16.7%)
VI. Malignant					
papillary carcinoma	1 (1.0%)	0 (0%)	0 (0%)	0 (0%)	1 (16.7%)
Total	104	42	24	32	6

FNAC, fine needle aspiration cytology; AUS, atypia of undetermined significance; CA, cytologic atypia, AA, architectural atypia; H, Hurthle cell; FN, follicular neoplasm; FA, follicular adenoma; FTC, follicular thyroid carcinoma; HA, Hurthle cell adenoma; HC, Hurthle cell carcinoma.

**Table 2:** Revised thyroid FNAC diagnostic categories of histologically proven follicular adenoma and carcinoma, and Hurthle cell adenoma and carcinoma.

FNAC diagnostic category	No. of surgical specimens	Final diagnosis			
		FA	FTC	HA	HC
I. Non-diagnostic	20 (19.2%)	9 (21.4%)	4 (16.7%)	7 (21.9%)	0 (0%)
II. Benign	8 (7.7%)	3 (7.1%)	2 (8.3%)	3 (9.4%)	0 (0%)
III. AUS	60 (57.7%)	22 (52.4%)	14 (58.3%)	19 (59.4%)	5 (83.3%)
AUS-CA	16 (15.4%)	7 (16.7%)	4 (16.7%)	5 (15.6%)	0 (0%)
AUS-AA	19 (18.3%)	10 (23.8%)	8 (33.3%)	1 (3.1%)	0 (0%)
AUS-CA and AA	7 (6.7%)	4 (9.5%)	3 (12.5%)	0 (0%)	0 (0%)
AUS-H	19 (18.3%)	1 (2.4%)	0 (0%)	13 (40.6%)	5 (83.3%)
IV. Suspicious for a FN	16 (15.4%)	8 (19%)	4 (16.7%)	3 (9.4%)	1 (16.7%)
V. Suspicious for malignancy					
suspicious for papillary carcinoma	0 (0%)	0 (0%)	0 (0%)	0 (0%)	0 (0%)
VI. Malignant					
papillary carcinoma	0 (0%)	0 (0%)	0 (0%)	0 (0%)	0 (0%)
Total	104	42	24	32	6

FNAC, fine needle aspiration cytology; AUS, atypia of undetermined significance; CA, cytologic atypia, AA, architectural atypia; H, Hurthle cell; FN, follicular neoplasm; FA, follicular adenoma; FTC, follicular thyroid carcinoma; HA, Hurthle cell adenoma; HC, Hurthle cell carcinoma.

**Fig 4:**
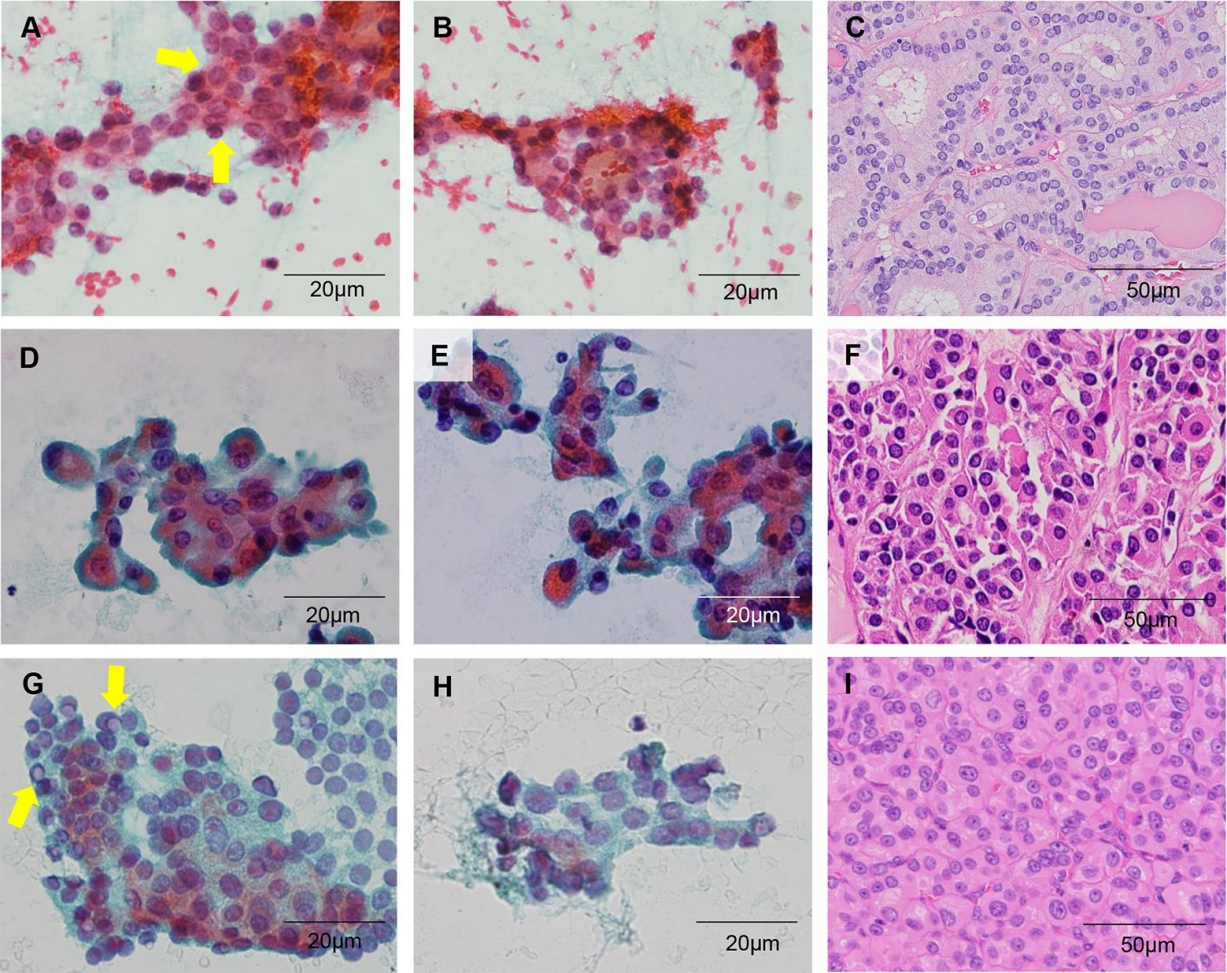
Representative cytologic and histologic features of misdiagnosed cases. (A-C) Case No. 50. (A) Nuclear atypia including chromatin clearing and nuclear groove (arrows) is present focally. (B) Microfollicles are focally noted. (C) Histologic features are consistent with follicular adenoma. (D-F) Case No.103. (D and E) Mild chromatin clearing and focal nuclear grooves are present. (F) Resected specimen reveals Hurthel cell carcinoma. (G-I) Case No. 27. (G) Artifacts mimicking intranuclear pseudoinclusion are noted. (H) However, the sample is entirely composed of Hurthle cells, and unequivocal nuclear atypia is absent. (I) Resection specimen reveals Hurthle cell carcinoma. (A, B, D, E, G, H; x400; scale bar, 20 μm) (C, F, I; x200; scale bar, 50 μm).

The summary of 20 cases with revised FNAC diagnoses is shown in Table 3. A total of 12 cases were originally underdiagnosed and were re-diagnosed into higher DCs. Three cases with original DC II (benign) were revised to DC III (AUS) due to the presence of architectural alteration including microfollicles and predominance of Hurthle cells in spite of low cellularity. Nine cases with original DC III (AUS) were re-categorized into DC IV (SFN) since all of these specimens showed at least moderate cellularity with prominent architectural alteration. The original diagnoses of suspicious for PTC and PTC were given in 2 cases and 1 case respectively, which were re-categorized as DC III (AUS) and IV (SFN). Of these 3 cases, 2 cases were proven to be HC. In the remaining one case (case No. 50) with final surgical diagnosis of FA, 3-dimensional branching sheet with focal nuclear enlargement and grooves might have led to the original FNAC DC V (suspicious for PTC) (Fig 4). Nevertheless, the diagnosis was revised to DC III (AUS) based on the moderate cellularity with focal architectural alteration, and the presence of equivocal nuclear atypia in a limited area (Fig 4).

**Table 3:** Summary of the thyroid FNACs with revised diagnosis upon review.

Case No.	Original diagnosis	Revised diagnosis	Final surgical diagnosis	Reason for revision
71	Benign	AUS-H	HA	predominantly Hurthle cells, no colloid, architectural alteration
73	Benign	AUS-MF	FTC	moderately cellular specimen with abundant microfollicles
76	Benign	Non-diagnostic	HA	less than 60 follicular cells, no colloid
78	Benign	AUS-MF	FA	less than 60 follicular cells with predominant microfollicles, no colloid
99	Benign	Non-diagnostic	FTC	less than 60 follicular cells, no colloid
54	AUS-CA	SFN	FA	moderately cellular specimen with microfollicles, trabecular pattern, transgressing vessels
72	AUS-CA	SFN	FA	moderately cellular specimen with abundant microfollicles
86	AUS-CA	AUS-H	HA	less than 60 Hurthle cells with architectural alteration, no colloid
20	AUS-AA	SFN	FA	moderately cellular specimen with abundant microfollicles, transgressing vessels
35	AUS-AA	SFN	FTC	moderately cellular specimen with abundant microfollicles
57	AUS-AA	SFN	FA	moderately cellular specimen with abundant microfollicles, transgressing vessels
61	AUS-AA	SFN	FA	moderately cellular specimen with abundant microfollicles, transgressing vessels
60	AUS-H	SFN	HA	moderately cellular specimen with Hurthle cells, large cell dysplasia, architectural alteration, transgressing vessels
67	AUS-H	SFN	HA	moderately cellular specimen with Hurthle cells, large cell dysplasia, architectural alteration, transgressing vessels
68	AUS-H	SFN	HA	moderately cellular specimen with Hurthle cells, abundant microfollicles, transgressing vessels
75	AUS-H	AUS-CA and AA	FA	barely over 60 follicular cells, focal Hurthle cell change, focal nuclear atypia, microfollicles
64	SFN	AUS-H	HA	moderately cellular specimen with Hurthle cells, predominantly macrofollicles, focal microfollicles
50	s/f PTC	AUS-CA and AA	FA	moderately cellular specimen with both macro- and microfollicles, focal nuclear atypia
103	s/f PTC	SFN	HC	moderately cellular specimen with Hurthle cells, large cell dysplasia, dissociated cells, trabecular pattern, transgressing vessels
27	PTC	AUS-H	HC	less than 60 Hurthle cells, large cell dysplasia, mild architectural alteration

FNAC, fine needle aspiration cytology; AUS, atypia of undetermined significance; SFN, suspicious for a follicular neoplasm, CA, cytologic atypia, AA, architectural atypia; H, hurthle cell; PTC, papillary thyroid carcinoma; FA, follicular adenoma; FTC, follicular thyroid carcinoma; HA, Hurthle cell adenoma; HC, Hurthle cell carcinoma.

### Comparison of preoperative FNAC and CNB diagnostic categories

**Fig 5:**
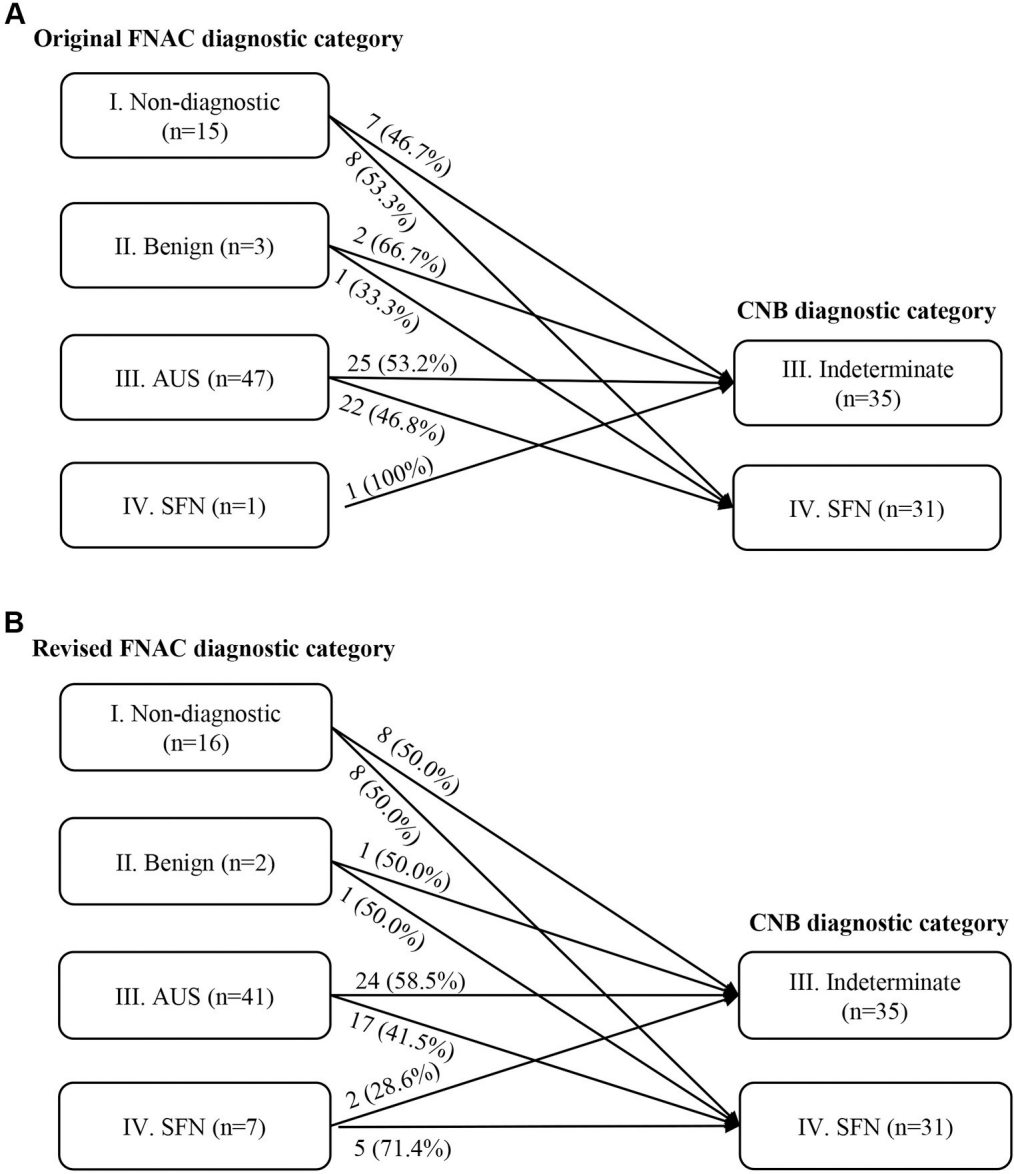
Comparison of Diagnostic Categories (DCs) of Fine Needle Aspiration Cytology (FNAC) and Core Needle Biopsy (CNB). (A) Among the 66 cases with both FNAC and CNB DCs available, 8 (53.3%), 1 (33.3%), and 22 (46.8%) cases with non-diagnostic, benign, and atypia of undetermined significance (AUS) DCs in original FNAC diagnoses were re-categorized as DC IV (suspicious for a follicular neoplasm) in CNB. (B) 8 (50.0%), 1 (50.0%), and 17 (41.5%) cases classified as non-diagnostic, benign, and AUS with the revised FNAC diagnoses were diagnosed as DC IV (suspicious for a follicular neoplasm) in CNB, suggesting under-diagnosis in FNAC.

Of the 104 cases, 66 cases were re-examined with thyroid CNB before surgical resection. They were initially categorized as non-diagnostic (n = 15), benign (n = 3), AUS (n = 47), and SFN (n = 1) in FNAC (Fig 5). In CNB, 8 (53.3%), 1 (33.3%), and 22 (46.8%) cases with the original FNAC DC I (non-diagnostic), DC II (benign), and DC III (AUS) were diagnosed as CNB DC IV (SFN). After review of original FNAC, 8 (50.0%), 1 (50.0%), and 17 (41.5%) cases with revised FNAC DC I (non-diagnostic), DC II (benign), and DC III (AUS) were diagnosed with DC IV (SFN) in CNB (Fig 5). The frequencies of DCs in the 66 CNB are listed in Table 4. In total, 35 (53.0%) were categorized as indeterminate lesion and 31 (47.0%) were classified as SFN in CNB. There were no cases with non-diagnostic or benign categories. None of the cases were categorized as suspicious for PTC or PTC.

**Table 4:** Thyroid CNB diagnostic category of histologically proven follicular adenoma and carcinoma, and Hurthle cell adenoma and carcinoma.

CNB diagnostic category	No. of surgical specimen	Final diagnosis			
		FA	FTC	HA	HC
I. Nondiagnostic	0 (0%)	0 (0%)	0 (0%)	0 (0%)	0 (0%)
II. Benign lesion	0 (0%)	0 (0%)	0 (0%)	0 (0%)	0 (0%)
III. Indeterminate lesion	35 (53.0%)	16 (53.3%)	9 (60.0%)	8 (42.1%)	2 (100.0%)
IIIA. Indeterminate lesion with NA	0 (0%)	0 (0%)	0 (0%)	0 (0%)	0 (0%)
IIIIB. Indeterminate lesion with AA	35 (53.0%)	16 (53.3%)	9 (60.0%)	8 (42.1%)	2 (100.0%)
IV. Suspicious for a follicular neoplasm (SFN)					
IVA. SFN without NA	30 (45.5%)	13 (43.3%)	6 (40.0%)	11 (57.9%)	0 (0%)
IVB. SFN with NA	1 (1.5%)	1 (3.3%)	0 (0%)	0 (0%)	0 (0%)
V. Suspicious for malignancy	0 (0%)	0 (0%)	0 (0%)	0 (0%)	0 (0%)
VI. Malignant	0 (0%)	0 (0%)	0 (0%)	0 (0%)	0 (0%)
Total No.	66	30	15	19	2

CNB, core needle biopsy; NA, nuclear atypia, AA, architectural atypia; FA, follicular adenoma; FTC, follicular thyroid carcinoma; HA, Hurthle cell adenoma; HC, Hurthle cell carcinoma.

### Comparison of cytologic features according to FNAC diagnostic categories

Various cytologic parameters including cellularity, presence of artifact, architectural pattern, nuclear features, and background quality were analyzed in FNAC slides and were compared among cases with different DCs (Table 5). Compared to FNACs with DC II (benign), those with DC IV (SFN) showed a significant association with higher cellularity (p<0.001). In terms of architectural pattern, predominant microfollicular pattern, presence of architectural crowding, 3-dimensional branching pattern, microfollicles arranged in trabecular pattern, and transgressing vessels were more commonly identified in the specimens with DC IV (SFN) (all p<0.05). Nuclear enlargement (p = 0.007) was more frequent in the DC IV (SFN) cases while the frequency of anisonucleosis, nuclear hyperchromasia, prominent nucleoli, small cell dysplasia, and large cell dysplasia did not differ between the two groups. In addition, the presence of background colloid, especially watery colloid, was associated with DC II (benign) (p = 0.032) (Table 5).

**Table 5:** Comparison of cytological features according to FNAC diagnostic category.

Cytological features	Benign	AUS	SFN	P-value^a^	P-value^b^
**Cellularity**				<0.001	0.008*
<60	6 (75.0%)	17 (28.3%)	0 (0%)		
Barely over 60	2 (25.0%)	19 (31.7%)	3 (18.8%)		
Moderately to markedly cellular	0 (0%)	24 (40.0%)	13 (81.3%)		
**Artifact (drying, clotting, etc.)**	3 (37.5%)	42 (70.0%)	10 (62.5%)	0.390	0.566
**Macrofollicle**				<0.001	<0.001
None to focal	2 (25.0%)	53 (88.3%)	16 (100.0%)		
Predominant	6 (75.0%)	7 (11.7%)	0 (0%)		
**Microfollicle**				0.001	0.002
None to focal	8 (100.0%)	42 (70.0%)	4 (25.0%)		
Predominant	0 (0%)	18 (30.0%)	12 (75.0%)		
**Microfollicles arranged in trabecular pattern**	0 (0%)	12 (20%)	8 (50.0%)	0.022	0.015
**Architectural crowding**	3 (37.5%)	59 (98.3%)	16 (100.0%)	0.001	1.000
**3-dimensional branching**	2 (25.0%)	42 (70.0%)	15 (93.8%)	0.001	0.058
**Trabecular or solid pattern**	0 (0%)	2 (3.3%)	3 (18.75%)	0.532	0.081
**Transgressing vessels**	1 (12.5%)	12 (20.0%)	11 (68.8%)	0.027	<0.001
**Nuclear enlargement**	3 (37.5%)	57 (95.0%)	15 (93.8%)	0.007	1.000
**Anisonucleosis**	4 (50.0%)	46 (76.7%)	9 (56.3%)	1.000	0.105
**Nuclear hyperchromasia**	2 (25.0%)	21 (35.0%)	5 (31.3%)	1.000	0.779
**Prominent nucleoli**	0 (0%)	11 (18.3%)	4 (25.0%)	0.262	0.724
**Small cell dysplasia**	0 (0%)	1 (1.7%)	0 (0%)	1.000	1.000
**Large cell dysplasia**	0 (0%)	8 (13.3%)	4 (25.0%)	0.262	0.265
**Nuclear chromatin clearing**	0 (0%)	25 (41.7%)	8 (50.0%)	0.022	0.55
**Nuclear groove**	1 (12.5%)	39 (65.0%)	8 (50.0%)	0.178	0.272
**Nuclear inclusion mimicker**	0 (0%)	5 (8.3%)	0 (0%)	1.000	0.578
**Blood**	4 (50.0%)	34 (56.7%)	9 (56.3%)	1.000	1.000
**Cystic change**	1 (12.5%)	9 (15.0%)	0 (0%)	0.333	0.191
**Colloid**				0.032	0.359
Absent	3 (37.5%)	46 (76.7%)	10 (62.5%)		
Thin colloid	3 (37.5%)	1 (1.7%)	0 (0%)		
Thick colloid	2 (25.0%)	11 (18.3%)	6 (37.5%)		
Thin and thick colloid	0 (0%)	2 (3.3%)	0 (0%)		
**Colloid quantity**				0.080	0.521
Focal	1 (12.5%)	13 (21.7%)	5 (31.3%)		
Prominent	4 (50.0%)	1 (1.7%)	1 (6.3%)		
Total	8	60	16		

AUS, atypia of undetermined significance; SFN, suspicious for a follicular neoplasm.

^a^Benign vs. SFN

^b^AUS vs. SFN.

Compared to those cases with DC III (AUS), cases diagnosed as DC IV (SFN) were associated with higher cellularity, predominant microfollicular pattern, microfollicles arranged in trabecular pattern, and transgressing vessels (all p<0.05) (Table 5).

### Correlation of FNAC diagnostic categories with histologic features in surgical resection specimens

We compared histologic features of tumor in the resection specimen among nodules with different FNAC DCs (Table 6). Among the various histologic features including proportion of normo- and macrofollicular pattern, papillary hyperplasia, intratumoral fibrosis, calcification or bony metaplasia, cystic degeneration, hemorrhage, background lymphocytic thyroiditis, and tumor size, only the presence of normo- and macrofollicular pattern in ≥2/3 of the tumoral area was significantly associated with tumors diagnosed as DC II (benign) compared to those diagnosed as DC IV (SFN) in FNAC (p = 0.028). There were no significant differences in the other histologic variables between DC II (benign) and DC IV (SFN) or DC III (AUS) and DC IV (SFN) (Table 6).

**Table 6:** Comparison of histologic features of tumor in resection specimen according to FNAC diagnostic category.

Histologic features	Benign	AUS	SFN	P-value^a^	P-value^b^
**Normo-macrofollicular pattern ≥2/3**	3 (37.5%)	5 (8.3%)	0 (0%)	0.028	0.578
**Papillary hyperplasia**	1 (12.5%)	3 (5.0%)	2 (13.3%)	1.000	0.282
**Intratumoral fibrosis**	2 (25.0%)	33 (55.0%)	6 (40.0%)	0.667	0.191
**Calcification or bony metaplasia**	1 (12.5%)	3 (5.0%)	0 (0%)	0.333	1.000
**Cystic degeneration**	2 (28.6%)	17 (28.3%)	4 (26.7%)	1.000	1.000
**Background lymphocytic thyroiditis**	2 (28.6%)	8 (13.3%)	4 (26.7%)	1.000	0.265
**Size of tumor, median (range, cm)**	1.0 (0.5–5.2)	2.3 (0.6–6.5)	2.4 (0.9–5.5)	0.238	0.954
**USG findings**				0.128	0.092
Low	1 (14.3%)	11 (18.3%)	0 (0%)		
Indeterminate	6 (85.7%)	38 (63.3%)	15 (100.0%)		
Suspicious	1 (14.3%)	2 (3.3%)	0 (0%)		
NA	0 (0%)	9 (15%)	1 (6.7%)		
Total	8	60	16		

AUS, atypia of undetermined significance; SFN, suspicious for a follicular neoplasm; USG, ultrasonography.

^a^Benign vs. SFN

^b^AUS vs. SFN.

### Comparison of cytologic features of histologically proven benign and malignant neoplasm

We also compared the cytologic parameters of nodules with the final surgical diagnoses of benign and malignant tumors (Table 7). FNACs of FTC were more cellular (p<0.001) and more commonly showed microfollicle arranged in trabecular pattern (p = 0.042) than FA. Compared with HAs, prominent nucleoli (p = 0.001) and large cell dysplasia (p = 0.007) were observed more often in HCs.

**Table 7:** Comparison of cytological features between histologically proven benign and malignant neoplasms.

Cytological features	FA	FTC	HA	HC	P-value^a^	P-value^b^
**Cellularity**					<0.001	0.168
<60	10 (30.3%)	8 (40.0%)	4 (16.0%)	1 (16.7%)		
Barely over 60	10 (30.3%)	1 (5.0%)	11 (44.0%)	2 (33.3%)		
Moderately cellular	13 (39.4%)	11 (55.0%)	10 (40.0%)	3 (50.0%)		
**Artifact (drying, clotting, etc.)**	20 (60.6%)	11 (55.0%)	20 (80.0%)	4 (66.7%)	0.688	0.596
**Macrofollicle**					0.457	0.553
None to focal	29 (87.9%)	16 (80.0%)	20 (80.0%)	6 (100.0%)		
Predominant	4 (12.1%)	4 (20.0%)	5 (20.0%)	0 (0%)		
**Microfollicle**					0.374	0.553
None to focal	19 (57.6%)	9 (45.0%)	20 (80.0%)	6 (100.0%)		
Predominant	14 (42.4%)	11 (55.0%)	5 (20.0%)	0 (0%)		
**Microfollicle arranged in trabecular pattern**	5 (15.2%)	8 (40.0%)	4 (16.0%)	1 (16.7%)	0.042	1.000
**Architectural crowding**	30 (90.9%)	18 (90.0%)	24 (96.0%)	6 (100.0%)	1.000	1.000
**3-dimensional branching**	22 (66.7%)	13 (65.0%)	19 (76.0%)	5 (83.3%)	0.901	1.000
**Trabecular or solid pattern**	1 (3.0%)	0 (0%)	3 (12.0%)	1 (16.7%)	1.000	1.000
**Transgressing vessels**	8 (24.2%)	2 (10.0%)	12 (48.0%)	2 (33.3%)	0.286	0.664
**Nuclear enlargement**	27 (81.8%)	17 (85.0%)	25 (100.0%)	6 (100.0%)	0.767	1.000
**Anisonucleosis**	20 (60.6%)	11 (55.0%)	22 (88.0%)	6 (100.0%)	0.688	1.000
**Nuclear hyperchromasia**	3 (9.1%)	5 (25.0%)	14 (56.0%)	6 (100.0%)	0.137	0.066
**Prominent nucleoli**	1 (3.0%)	3 (15.0%)	5 (20.0%)	6 (100.0%)	0.145	0.001
**Small cell dysplasia**	NE	NE	1 (4%)	0 (0%)	-	1.000
**Large cell dysplasia**	NE	NE	5 (20.0%)	5 (83.3%)	-	0.007
**Nuclear chromatin clearing**	17 (51.5%)	8 (40.0%)	5 (20.0%)	1 (16.7%)	0.416	1.000
**Nuclear groove**	20 (60.6%)	11 (55.0%)	14 (56.0%)	3 (50.0%)	0.688	1.000
**Nuclear inclusion mimicker**	2 (6.1%)	0 (0%)	2 (8.0%)	1 (16.7%)	0.521	0.488
**Blood**	18 (54.5%)	7 (35.0%)	18 (72.0%)	4 (66.7%)	0.167	1.000
**Cystic change**	3 (9.1%)	1 (5.0%)	4 (16.0%)	2 (33.3%)	1.000	0.567
**Colloid**					0.446	0.315
Absent	24 (72.7%)	14 (70.0%)	15 (60.0%)	6 (100.0%)		
Thin colloid	2 (6.1%)	0 (0%)	2 (8.0%)	0 (0%)		
Thick colloid	7 (21.2%)	6 (30.0%)	6 (24.0%)	0 (0%)		
Thin and thick colloid	0 (0%)	0 (0%)	2 (8.0%)	0 (0%)		
**Colloid quantity**					1.000	-
Focal	8 (24.2%)	5 (25.0%)	8 (32.0%)	NE		
Prominent	1 (3.0%)	1 (5.0%)	2 (8.0%)	NE		
Total	33	20	25	6		

FA, follicular adenoma; FTC, follicular thyroid carcinoma; HA, Hurthle cell adenoma; HC, Hurthle cell carcinoma; NE, not evaluable.

^a^FA vs. FTC

^b^HA vs. HC.

## Discussion

The DC IV (SFN) in TBSRTC had been referred to various terminologies, including “follicular lesion”, “follicular proliferation”, “SFN”, or “FN” prior to implementation of TBSRTC [25–29]. This reflects the difficulty in differentiating FACHACs from other follicular proliferative lesions such as cellular adenomatoid nodule, follicular variant of PTC, and NIFTP by FNAC alone due to their similar cytologic features [3–5]. The goal of designating TBSRTC DC IV (SFN) is not to seek out all FACHACs but to identify those thyroid nodules with malignant potential. Currently, TBSRTC DC IV (SFN) is recommended for nodules that are at least moderately cellular and show prominent architectural alteration including cellular crowding, microfollicles, and dispersed isolated cells [1, 2]. Nevertheless, many researchers have reported poor cytohistologic correlation of DC IV (SFN) [22, 27, 30–33].Up to 44% of the nodules of which the cytologic features were concordant with DC IV (SFN), turned out to be non-neoplastic lesions [27], implying that diagnosing follicular-patterned neoplasms remains challenging.

In our institution, DC IV (SFN) constituted only 0.8% of all FNAC cases diagnosed between 2012 and 2014 [34]. Considering the much lower incidence of FTC as opposed to PTC in Korea compared to western countries, the diagnostic rate of DC IV (SFN) can be assumed to be low in Korea. We tried to uncover whether there are other reasons for low rate of DC IV (SFN) than low incidence of FTC in our institute.

In the present study, 20 out of 104 cases were re-categorized after a thorough review of the FNAC slides. A total of 3 cases-1 FA and 2 HCs- were originally misdiagnosed as suspicious for PTC or PTC. Overestimation of mild nuclear atypia, especially in Hurthle cells, was the main cause of these misdiagnoses. The extremely low incidence of HNs compared with PTC in our institution might have affected the misdiagnoses as well. Although nuclear grooves, intranuclear inclusions, and even slight chromatin clearing can be seen in HNs, nuclei are generally round with prominent nucleoli [35–37]. Therefore, cytopathologists should always be aware of this pitfall and be careful not to misdiagnose HNs as PTC since treatment options including lymph node dissection and prognosis can be different in these two diseases [38, 39].

Of the 11 cases with DC III (AUS) in original FNAC diagnoses, 9 cases fulfilled the criteria of DC IV (SFN). 3 out of 5 cases with the original DC II (benign) were consistent with DC III (AUS). This under-diagnosis might have resulted from some confounding factors such as the pathologist’s reluctance in diagnosing DC IV (SFN) due to fear of diagnostic lobectomy and lack of experience in thyroid cytopathology. Although we have revised DCs in 20 cases, over half of DC I (non-diagnostic) cases in FNAC were categorized as DC IV (SFN) in CNB, suggesting problems in specimen inadequacy of FNAC specimens. Moreover, significant proportions of DC II (benign) and DC III (AUS) cases were diagnosed as DC IV in CNB, suggesting under-diagnosis in FNAC. The advantage of CNB over FNAC in diagnosing FNs has also been demonstrated by other researchers, and it might be due to the fact that thyroid CNB not only reveals the nodule itself but also its histologic relationship with the capsule and adjacent normal thyroid tissue [13–15]. This suggests that thyroid CNB can be useful primary or complementary tool when FACHAC is suspected clinically or radiologically.

Apart from the innate disadvantage of FNAC in diagnosing FACHAC as opposed to CNB, we further analyzed diverse cytomorphologic and histologic parameters of each nodule, and we investigated the differences between the groups with DC II (benign), III (AUS) and IV (SFN) in FNAC to identify other possible reasons for under-diagnosis. When we analyzed the cytologic parameters, the cases that had been underdiagnosed were far less cellular than those with DC IV(SFN), reflecting the significance of acquiring a proper FNAC specimen with sufficient cellularity. In addition, a predominantly macrofollicular pattern and the presence of thin colloid were associated with under-diagnosis. Similarly, a predominant normo- or macrofollicular pattern in surgically resected specimens was associated with under-diagnosis. Our result is in line with previous studies which have demonstrated that macrofollicular variant of FAs and FTCs can mimic nodular hyperplasia and are generally underappreciated in FNAC [4, 40–42]. Bongiovanni et al. have recently described the presence of pathogenic somatic mutations in *DICER1*, *EIF1AX*, and *DNMT3A* genes in four cases of macrofollicular variant of FTC, suggesting that additional molecular testing can be helpful in a clinically malignant nodule with deceptively benign FNAC findings [40]. Further investigations are warranted to determine whether a certain mutation is more common in macrofollicular variant of FTC.

Finally, we compared the cytologic features between the nodules finally diagnosed as benign versus malignant neoplasm (FA versus FTC, and HA versus HC). Compared with FAs, FTCs were significantly more hypercellular. Although controversial, cellularity has been proposed as one of the distinguishing features of malignancy in some studies [43, 44]. Of note, microfollicles were sometimes arranged in trabecular or branching pattern, which was more frequently identified in FTCs (40.0%) than FAs (15.2%). Han et al. have also described that this trabecular pattern was more commonly observed in FAs and FTCs than in nodular hyperplasia although the frequency did not differ between FAs and FTCs [22]. Further evaluation in a larger cohort is required to elucidate the significance of this unique pattern. Other cytologic parameters reported to be associated with malignancy include absence of thin colloid, absence of macrofollicular pattern, crowding, high nuclear-cytoplasmic ratio, nuclear atypia, and transgressing vessels [43–46], which were not found to be significantly different between the two groups. Recently, Gupta et al. [47] and Savala et al. [48] applied artificial neural network models and gray level co-occurrence of matrix method to analyze basic cytomorphological features and they reported promising results suggesting that objective measurement can be a solution to more accurate diagnosis.

When comparing HCs with HAs, HCs more frequently showed prominent nucleoli and large cell dysplasia. Similarly, Renshaw et al. have suggested that the presence of at least one of small cell dysplasia, large cell dysplasia, crowding, or dyshesion favors malignancy [23, 49]. In addition, four characteristic cytologic features of HCs including syncytial fragments, small sized cells with high nucleo-cytoplasmic ratio, prominent nucleoli, and intranuclear inclusions have been described [45]. Although others have disputed consistency, these morphologic criteria combined with clinical parameters and recently developed molecular testing may be helpful for accurate triage of HNs [50, 51].

Interestingly, we identified transgressing vessels not only in HNs but also in conventional FAs and FTCs although the frequency did not significantly differ between FAs and FTCs. The presence of transgressing vessels has been a traditional parameter that favors HNs over non-neoplastic Hurthle cell proliferative lesions associated with lymphocytic thyroiditis or Graves’ disease [29, 36, 45, 52]. Lubitz et al. have described this structure in FAs and FTCs in addition to HNs and reported that the presence of transgressing vessels was associated with malignancy [43]. Unfortunately, we were unable to elucidate the association of transgressing vessels with neoplasm, because we did not include non-neoplastic lesions in the current study. Further investigation including non-neoplastic lesion would be needed.

In the present study, we performed a systematic and comprehensive investigation of fundamental cytomorphologic and histologic parameters of histologically proven FACHACs. We observed that there was a significant number of mis- or underdiagnoses in preoperative FNAC of FACHAC. Overestimation of the subtle nuclear atypia was responsible for most of the misdiagnoses. As for underdiagnoses, the innate cytohistologic features as well as inadequate sampling were the leading causes. Of note, we identified some cytologic features associated with malignancy. Since application of recently developed molecular testing and artificial intelligence are not feasible in routine practice at most institutions, understanding the basic cytologic features along with complementary CNB should improve the diagnostic accuracy of FACHAC in FNAC.

## Conclusions

In conclusion, it is not only the low incidence, but also the sample quality and innate cytohistological features of a tumor that are associated with under-interpretation of FACHAC in FNAC. To improve sensitivity and specificity, acquiring a proper sample is a prerequisite along with cautious interpretation of basic cytologic features including nuclear atypia. Higher cellularity, the presence of microfollicles arranged in trabecular pattern, nucleolar prominence, and the presence of large cell dysplasia can be helpful in differentiating FTC or HC from FA or HA.

## Supporting information

S1 Data Summary of cytologic and histologic features of follicular adenoma and carcinoma, Hurthle cell adenoma and carcinoma included in this study. (XLSX)
